# Direct detection of *Corynebacterium striatum*, *Corynebacterium propinquum*, and *Corynebacterium simulans* in sputum samples by high-resolution melt curve analysis

**DOI:** 10.1186/s12879-020-05633-z

**Published:** 2021-01-07

**Authors:** Shuai Xu, Xiaotong Qiu, Xuexin Hou, Haijian Zhou, Dongke Chen, Xuebing Wang, Lichao Han, Dan Li, Lina Sun, Xingzhao Ji, Minghui Li, Jingshan Zhang, Mengtong Li, Zhenjun Li

**Affiliations:** 1 State Key Laboratory for Infectious Disease Prevention and Control, National Institute for Communicable Disease Control and Prevention, Chinese Center for Disease Control and Prevention, Beijing, China; 2 Department, Beijing Hospital, National Center of Gerontology, Institute of Geriatric Medicine, Chinese Academy of Medical Sciences, Beijing, People’s Republic of China

**Keywords:** High-resolution melt curve analysis, *Corynebacterium* species, Bacterial identification, Diagnostics

## Abstract

**Background:**

Pulmonary infections caused by non-diphtheriae corynebacteria are increasing. However, rapid identification of *Corynebacterium* species poses a challenge due to the low genetic variation within the genus.

**Methods:**

Three reference strains and 99 clinical isolates were used in this study. A qPCR followed by high-resolution melting (HRM) targeting *ssrA* was performed to simultaneously identify *C. striatum*, *C. propinquum* and *C. simulans*. To further evaluate this assay’s performance, 88 clinical sputum samples were tested by HRM and the detection results were compared with those of the traditional culture method and multiple cross-displacement amplification (MCDA) assay.

**Results:**

The melting curve produced by a pair of universal primers generated species-specific HRM curve profiles and could distinguish the three target species from other related bacteria. The limit of detection of HRM assay for DNA from the three purified *Corynebacterium* species was 100 fg. Compared with the culture method, HRM detected 22 additional positive specimens, representing a 23.9% relative increase in detection rate. The HRM assay had 98.4% (95% confidence interval [CI], 90.5–99.9%) sensitivity and 100% (95% CI, 82.8–100%) specificity. Additionally, 95.5% concordance between HRM and MCDA (κ = 0.89 [95% CI, 0.79–0.99]) was noted.

**Conclusions:**

The HRM assay was a simple, rapid, sensitive, and specific diagnostic tool for detecting *C. striatum*, *C. propinquum,* and *C. simulans*, with the potential to contribute to early diagnosis, epidemiological surveillance, and rapid response to outbreak.

**Supplementary Information:**

The online version contains supplementary material available at 10.1186/s12879-020-05633-z.

## Background

The genus *Corynebacterium* is composed of aerobic, non–spore-forming, pleomorphic, Gram-positive bacilli with worldwide distribution. The most well-established and well-described pathogen in this genus, *C. diphtheria*, is the main causative agent of diphtheria, the incidence of which has dropped due to effective vaccination programs [1]. However, in recent years, there has been a considerable increase in reports of non-diphtheriae *Corynebacterium* species, which have been linked to multiple hospital outbreaks and nosocomial infections [2–4]. Although these microorganisms are common components of the skin microbiota and mucous membranes, their clinical significance as emerging respiratory pathogens has been demonstrated by various studies [4–6]. Of note, recent reports show that multidrug-resistant strains of the species *C. striatum* are emerging rapidly [7–10]. Early detection and identification of *Corynebacterium* species are essential to intervention and infection treatment efforts.

Traditionally, these microorganisms have been routinely identified by biochemical tests using the API Coryne system (bioMérieux, Craponne, France) or the RapID CB PLUS system (Thermo Fisher Scientific, Waltham, MA, USA) in clinical microbiology laboratories [11, 12]. However, these methods have low sensitivity, and are time-consuming and unreliable for species identification, especially in the case of *C. simulans*, due to its similarities with *C. striatum* [13, 14]. Identification by 16S ribosomal ribonucleic acid (rRNA) and *rpoB* gene sequencing produces more reliable results, but is slow and cost prohibitive in developing countries [15, 16]. Matrix-assisted laser desorption/ionization–time-of-flight mass spectrometry (MALDI-TOF MS) has been applied to perform accurate species-level identification of *Corynebacterium* spp. clinical isolates, but this technology is not yet fully accessible to clinical microbiology laboratories in resource-limited settings [17]. Also, MALDI-TOF requires pure cultures as starting material, which precludes rapid diagnosis. Therefore, suitable detection assays that are rapid, reliable, and cost-effective are always in demand for effective control and treatment strategies against infections caused by emerging *Corynebacterium* species.

The high-resolution melting (HRM) assay, a recently developed technique based on quantitative real-time polymerase chain reaction (qPCR) that detects genetic variation in deoxyribonucleic acid (DNA) sequences, provides a good alternative for molecular diagnosis [18]. Before HRM analysis can be performed, the region of interest is amplified using PCR in the presence of a fluorescent dye (EvaGreen) that is homogenously intercalated into the double-stranded (ds) DNA. After PCR, the amplicon is gradually heated at increasing temperatures, and the ds PCR product denatures into two single strands, releasing the binding dye and leading to a decrease in fluorescence level. The rate of dissociation of the amplicon mainly depends on GC-content, sequence length, complementarity, and nearest-neighbor thermodynamics [19]. A specific and characteristic melting profile can be produced for the amplicon by monitoring changes in fluorescence intensity. The HRM assay is an easily implemented, closed-tube method that can simultaneously detect closely related species within approximately 2 h. In addition, it is not restricted to cultured material, but can detect DNA extracted directly from clinical specimens. The HRM technique has been successfully employed in concurrently identifying and differentiating between several pathogens, such as bacteria, viruses, and fungi [20–23].

In this study, we report the development of a qPCR–based HRM assay capable of detecting *C. striatum*, *C. propinquum,* and *C. simulans*, as well as distinguishing between them in pure cultures and clinical specimens with increased specificity and sensitivity.

## Methods

### Bacterial strains

Three reference strains (*C. striatum* ATCC 6940, *C. propinquum* DSM 44285, and *C. simulans* DSM 44415) and 99 clinical isolates were used in this study (Additional file 1). All clinical isolates were recovered from pulmonary specimens (80 sputum samples, 14 tracheal aspirates, and 5 bronchoalveolar lavage fluid samples) of patients clinically suspected of having respiratory tract infections, and were collected from 2016 to 2018 in the Department of Clinical Laboratory Medicine, Peking University People’s Hospital, Beijing, China. The organisms were identified by matrix-assisted laser desorption/ionization time-of-flight mass spectrometry (MALDI-TOF MS) using the Vitek MS (bioMérieux, France) system and the results were confirmed by 16S rRNA gene sequencing.

### Genomic DNA extraction

We extracted genomic DNA from reference strains and clinical isolates using QIAamp DNA Mini Kits (QIAGEN, Hilden, Germany) in accordance with the manufacturer’s instructions. DNA concentration and purity were measured using a spectrophotometer (NanoDrop ND-1000; Thermo Fisher) at A260/280. Purified DNA were kept at − 20 °C for subsequent experiments.

### Genomic target selection and primer design for HRM

**Fig. 1:**
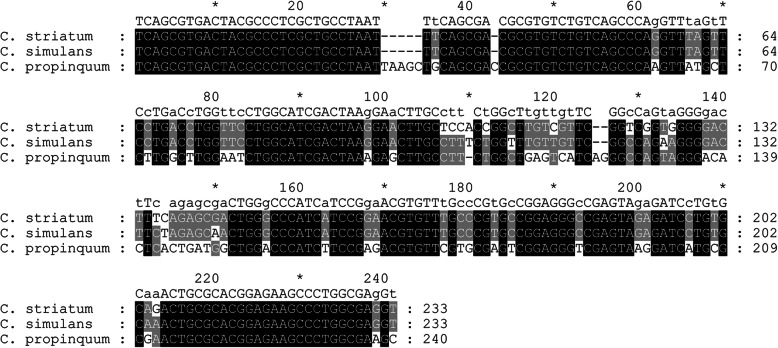
Sequence alignment of the *ssrA* from each *Corynebacterium* species. In the consensus line, capital letters represent conserved bases whereas lowercase letters represent substitutions or deletions. Gaps are shown as hyphens

As the *ssrA* gene, which encodes a transfer-messenger RNA (tmRNA), is highly conserved and phylogenetically informative [24], it was selected as the target. The sequences of *ssrA* loci for three reference strains were obtained from published genomes in GenBank (National Center for Biotechnology Information [NCBI], Bethesda, MA, USA; https://www.ncbi.nlm.nih.gov/genbank/) and aligned using SeqMan software (version 7.1.0; Fig. 1). In order to identify the best primer pair, 20 pairs of primers were designed based on the *ssrA* gene in the conserved region using CmSuite software version 8.0 (https://www.scied.com/pr_cmpro.htm). *C. striatum*, *C. propinquum,* and *C. simulans* were tested by PCR using 20 primer sets. The primers ssrA-Fwd (5′-TCAGCGTGACTACGCCCTC-3′) and ssrA-Rev (5′-RCYTCGCCAGGGCTTCTC-3′) displayed positive amplification and were selected for qPCR and HRM assays (data not shown).

### qPCR and HRM assays

A qPCR assay was set up containing the following components per reaction:

15 μl 2× Taqman PCR Master Mix (Thermo Fisher); 0.9 μl Primer F (10 μΜ); 0.9 μl Primer R (10 μΜ); 0.3 μl Rox Reference Dye II (100×); 1.5 μl EvaGreen, 20× in water (Biotium, Fremont, CA, USA); 1 ng DNA sample; and nuclease-free water to a total reaction volume of 30 μl. The qPCR assay was performed and validated using the ABI QuantStudio 6 Flex system (Thermo Fisher Scientific, USA) with the following run conditions: 1 cycle of 95 °C for 10 min, followed by 35 cycles of 95 °C for 15 s and 63 °C for 45 s. Next, HRM analysis was initiated by raising the temperature to 95 °C for 15 s and decreasing it to 60 °C for 1 min. Then, the melting curves were generated by increasing the temperature from 60 °C to 95 °C in increments of 0.025 °C/s. All of the amplicons were tested in triplicate to detect technical errors. The HRM data were analyzed using QuantStudio™ Real-Time PCR Software version 1.3. All of the experiments were performed in triplicate.

### Evaluation of the sensitivity of the HRM assay

To assess the sensitivity of the HRM assay for identification of *Corynebacterium* species, the genomic DNA templates of three reference strains were serially diluted with distilled water (10 ng, 100 pg, 10 pg, 1 pg, 100 fg, and 10 fg per μl) to define the limit of detection. DNA templates of *Nocardia farcinica* were used as the negative control, and distilled water was the blank control. Each DNA concentration was assayed in duplicate by HRM.

### Evaluation of the specificity of the HRM assay

To assess the specificity of the HRM assay, the PCR-HRM reactions were performed under the conditions described above with purely genomic DNA templates (10 ng/reaction) from 60 *C. striatum*, 3 *C. propinquum*, 6 *C. simulans,* and 30 non-target clinical samples (Additional file 1). All of the strains were cultured overnight on brain–heart infusion ager at 37 °C. Bacterial genomic DNA from all of the cultured strains were extracted using QIAamp DNA Mini Kits (Qiagen, Germany) in accordance with the manufacturer’s instructions. Analysis of each sample was carried out twice independently.

### Practical application of HRM in clinical sputum samples

In total, 88 human sputum samples were recovered from hospitalized patients with pulmonary infections. These samples were previously identified using the culture method and stored at − 70 °C until use in the HRM assay. To evaluate the practical application of the novel HRM method, we employed this assay in routine detection for 88 clinical sputum samples, and compared the results with those of the traditional culture method and the multiple cross displacement amplification (MCDA) assay developed in a previous study [25]. The DNA templates from the sputum samples were extracted using a Wizard® Genomic DNA Purification Kit (Promega, Fitchburg, WI, USA) in accordance with the manufacturer’s instructions. The extracted genomic DNA were used for HRM and MCDA tests. This experiment was carried out in duplicate independently.

### DNA sequencing

To further confirm the reliability of the HRM assay, all of the HRM-positive/culture-negative amplicons from qPCR-HRM were sent to Sangon Biotech (Shanghai, China) to undergo direct sequencing in both strands. In brief, the PCR amplicons produced from *ssrA* were purified with a QIAquick PCR purification kit (QIAGEN, Germany) and subjected to sequencing using the primers ssrA-Fwd and ssrA-Rev on the ABI PRISM 7500 Sequence Detection System (Applied Biosystems, USA). The sequencing results were analyzed using SeqMan software, and were further compared with the reference samples using the Basic Local Alignment Search Tool (BLAST; NCBI; https://blast.ncbi.nlm.nih.gov/Blast.cgi).

### Statistical analysis

To analyze assay sensitivity and specificity, we used the conventional culture method as the gold standard for the culture-positive specimens, while using consensus results from both the HRM and MCDA assays as a reference standard for the remaining specimens. The sensitivities of the HRM and MCDA assays were determined by the following equation:

$$ \mathrm{number}\ \mathrm{of}\ \mathrm{true}\ \mathrm{positive}/\left(\mathrm{number}\ \mathrm{of}\ \mathrm{true}\ \mathrm{positive}+\mathrm{number}\ \mathrm{of}\ \mathrm{false}\ \mathrm{negative}\right)\times 100\%. $$

Specificity was determined by the following equation [26]:

$$ \mathrm{number}\ \mathrm{of}\ \mathrm{true}\ \mathrm{negative}/\left(\mathrm{number}\ \mathrm{of}\ \mathrm{true}\ \mathrm{negative}+\mathrm{number}\ \mathrm{of}\ \mathrm{false}\ \mathrm{positive}\right)\times 100\%. $$

Additionally, 95% confidence intervals (CIs) were calculated for sensitivity and specificity using the Wilson score method. We used McNemar’s chi-square test to assess whether the performances of these tests were different. A *P* < 0.05 was considered to indicate statistical significance. The agreement levels between HRM and MCDA results were measured using Cohen’s kappa test, which was calculated as described previously [27].

## Results

### HRM analysis for identification of *C. striatum*, *C. propinquum*, and *C. simulans*

**Fig. 2:**
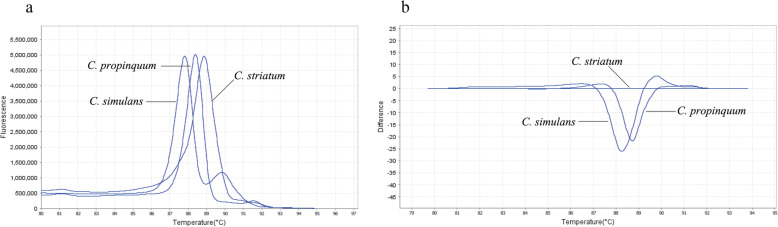
Representative HRM analysis for differentiating *Corynebacterium* species. **a** Normalized melting curves; **b** difference plot using *C. striatum* as the baseline

A pair of universal primers (ssrA-Fwd and ssrA-Rev), designed to detect *C. striatum*, *C. propinquum*, and *C. simulans* in HRM analysis, amplified fragments of 233, 240, and 233 bp, respectively, for these species. In the HRM analysis, *C. striatum* and *C. propinquum* each had a single peak, while *C. simulans* produced two melting peaks (Fig. 2a). A distinct HRM peak was observed in the normalized melting curves, allowing them easy to distinguish from each other. The melting temperatures for *C. striatum*, *C. propinquum*, and *C. simulans* using HRM primers were 88.91 °C, 88.44 °C, and 87.86 °C, respectively. Specifically, a high-resolution difference plot was constructed using the curved shape of *C. striatum* as a baseline, which indicated that these three species were indeed different through the HRM curves (Fig. 2b).

### Limit of detection of the HRM assay

**Fig. 3:**
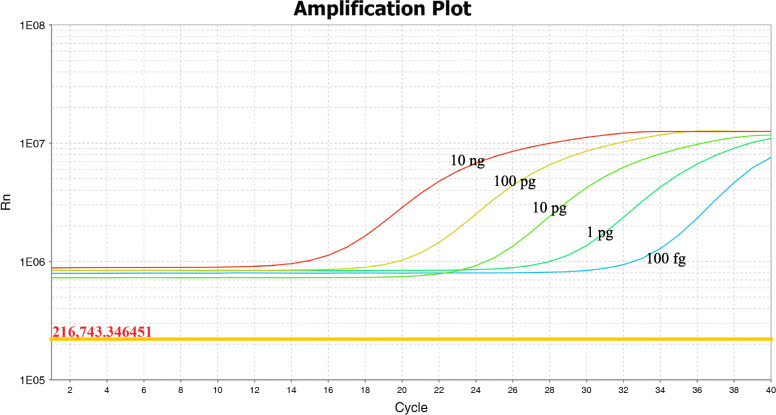
Limit of detection of the HRM assay

To assess the sensitivity of the HRM assay, serial dilutions (10 ng–10 fg) of total genomic DNA extracted from the three reference strains were subjected to a HRM assay. The decreasing concentrations of genomic DNA are presented from left to right in Fig. 3. The limit of detection of the HRM assay was 100 fg of genomic DNA per reaction for *C. striatum*, *C. propinquum*, and *C. simulans*.

### Analytical specificity of the HRM assay

To determine the specificity of the HRM assay, the purely genomic DNA extracted from 60 *C. striatum*, 3 *C. propinquum*, 6 *C. simulans,* and 30 non-target strains were tested using the HRM assay under the standard conditions described above. As expected, all 69 cultured samples belonging to *C. striatum*, *C. propinquum,* and *C. simulans* were amplified and formed unique and reproducible melting curves (Additional file 2). In contrast, the 30 non-target strains showed no melting curves. These results suggested that the HRM assay targeting *ssrA* identified *C. striatum*, *C. propinquum,* and *C. simulans* with 100% specificity.

### Evaluation of the HRM assay using clinical sputum samples

To further assess the suitability and usefulness of the HRM assay as a tool for *Corynebacterium* spp. detection, a total of 88 clinical human sputum samples were analyzed using the traditional culture method, a MCDA assay, and a HRM assay. The results are summarized in Table 1. In the 88 sputum samples, the culture produced 42 positives (47.7%), the MCDA produced 59 (67.0%), and the HRM assay produced 63 (71.6%).

**Table 1:** Identification of *Corynebacterium* species in clinical sputum samples by culture, MCDA assay, and HRM assay

	Culture	MCDA	HRM
*C. striatum*	39	59	60
*C. propinquum*	2	NA	2
*C. simulans*	1	NA	1
Total	42	59	63

*MCDA* multiple cross-displacement amplification, *HRM* high-resolution melting, *NA* No detection

Specimens were defined as true positives if: (i) *C. striatum*, *C. propinquum,* or *C. simulans* were recovered from the culture; or (ii) both MCDA and HRM results were positive for a culture-negative specimen [28–30]. Among the 42 culture-positive samples, the MCDA assay produced five false-negative results, while the HRM assay produced one (Table 2).

**Table 2:** Comparison of the HRM and MCDA assays with the culture method

		Culture		Total
		Positive	Negative	
MCDA	Positive	37	22	59
	Negative	5	24	29
HRM	Positive	41	22	63
	Negative	1	24	25
Total		42	46	88

*HRM* high-resolution melting, *MCDA* multiple cross-displacement amplification

Ultimately, 22 culture-negative specimens were found to be positive by the MCDA and HRM assays, results that were further confirmed by nucleic acid sequencing. These 22 specimens were considered true positives. Sensitivity was calculated to be 65.6% (95% CI, 52.6–76.7%) for the culture method, 92.2% (95% CI, 82.0–97.1%) for the MCDA assay, and 98.4% (95% CI, 90.5–99.9%) for the HRM assay; specificities were 100% (95% CI, 82.8–100%) for all three assays (Table 3). No significant differences in sensitivity or specificity were found between the MCDA and HRM assays (McNemar’s chi-square test, *P* > 0.5). However, the HRM assay was more sensitive than the culture method (*P* < 0.001), and 95.5% concordance between the MCDA assay and our HRM assay (κ = 0.89 [95% CI, 0.79–0.99]) was noted.

**Table 3:** Performance of the culture method, MCDA assay, and HRM assay in clinical sputum sample analysis

Method	TP	TN	FP	FN	Sensitivity (95% CI)	Specificity (95% CI)
Culture	42	24	0	22	65.6% (52.6–76.7)	100% (82.8–100)
MCDA	59	24	0	5	92.2% (82.0–97.1)	100% (82.8–100)
HRM	63	24	0	1	98.4% (90.5–99.9)	100% (82.8–100)

*TP* true positive, *TN* true negative, *FP* false positive, *FN* false negative, *CI* confidence interval, *MCDA* multiple cross-displacement amplification, *HRM* high-resolution melting

## Discussion

Due to being common components of the skin microbiota, non-diphtheriae *Corynebacterium* species are usually thought of as colonizers or contaminants [31, 32]. Because of this, as well as challenges in identification, they have not received a great deal of attention [33]. In recent years, the clinical relevance of these microorganisms has been recognized, particularly as a cause of respiratory-tract infections [5, 6, 34, 35]. A rapid, simple, specific, and sensitive molecular technique for identifying and differentiating between these species is essential for outbreak detection, epidemiological surveillance, and direct patient treatment, as most strains of *C. striatum* are resistant to multiple antimicrobials.

However, differentiation between these species remains difficult due to the low genetic variation between them. *C. striatum*, *C. propinquum*, and *C. simulans* have similar colony morphologies and cultural characteristics. *C. simulans* and *C. striatum* share high genetic homology, and their biochemical reactions are very similar [36]. Misidentification of *C. simulans* or *C. propinquum* as *C. striatum* by VITEK MS MALDI-TOF MS was observed in our investigation (data not shown), and this has also been reported previously [8]. Currently, few commercially available assays can rapidly differentiate between these species, and published assays rely on the use of pure cultures.

In this study, we developed a HRM assay that could differentiate between *C. striatum*, *C. propinquum,* and *C. simulans* in cultured samples and clinical specimens within approximately 2 h after DNA extraction. Analysis of the normalized melt curve produced with the universal primers generated species-specific HRM curve profiles. The three species were clearly differentiated by the melting temperature of the dissociation curves, with melting peaks at 88.91 °C for *C. striatum*, 88.44 °C for *C. propinquum,* and 87.86 °C for *C. simulans*. Furthermore, these three strains were successfully identified based on their different plots, which were mutually distinct.

We also evaluated the limit of detection of the newly established HRM assay. This assay could detect quantities as low as 100 fg using DNA from pure cultures as templates. Assay specificity was further evaluated by testing the genomic DNA of 69 corynebacteria clinical isolates and 30 non-target strains. Results showed that the high discriminatory power of the HRM assay developed for *C. striatum*, *C. propinquum,* and *C. simulans* also gave it the ability to specifically distinguish these three species from other related bacteria.

Furthermore, we showed that HRM could be applied to direct tests of clinical sputum samples. Among the 42 culture-positive samples, all were identified by HRM, while one false negative result occurred. This one sample was also found to be negative by the MCDA assay. This result suggested that DNA might have been lost or degraded during the preparation, processing, and storage of the sputum samples.

Furthermore, 22 samples were identified as negative by the culture method, but identified as positive by HRM and MCDA assays. In our study, the culture method was likely to miss one quarter (22/88) of *Corynebacterium* spp.-positive samples. Putative sensitivity increased from 65.6% using the culture method to 98.4% using the HRM assay. Compared with the culture method, the use of the HRM assay to detect the three *Corynebacterium* spp. could potentially improve overall sensitivity and reduce turnaround times.

Our HRM assay was also comparable to the MCDA method, showing a high concordance rate (95.5%) [25]. MCDA technology has revolutionized the detection of pathogens, but the established MCDA method can identify only one *Corynebacterium* species, *C. striatum*. In addition, this method requires the use of five pairs of primers, which can generate a complex mixture of various DNA products, and it can be difficult to distinguish between specific and non-specific products [37]. Compared with the MCDA assay, the HRM method we developed based on a pair of universal primers could specifically identify three *Corynebacterium* spp. in only one test.

In the present study, 95.3% of pulmonary infections were caused by *C. striatum,* 3.1% by *C. propinquum,* and 1.6% by *C. simulans*, as identified by HRM. Among members of *Corynebacterium* spp., *C. striatum* and *C. propinquum* have been recognized as pathogens of the respiratory tract, and have been found in cases of pneumonia and chronic obstructive pulmonary disease (COPD) in hospital settings [4, 38–40]. A comparative study noted that *C. striatum* was the most prevalent non-diphtheriae *Corynebacterium* found in sputum specimens, and found that it caused a large proportion of respiratory infections [1]. The high incidence of nosocomial outbreaks caused by *C. striatum* highlights the importance of routine species-level identification to avoid further spread and outbreaks [41].

Unlike *C. striatum* and *C. propinquum*, *C. simulans* has not been described as a cause of respiratory infection. Only a few well-documented infections have been reported, including one case of acute pyogenic spondylitis, one prosthetic joint infection, and one case of endocarditis [42–44]. The recovery of *C. simulans* from a respiratory specimen here appears to be novel and has clinical relevance, emphasizing its potential role as a causative pathogen of respiratory tract infections.

There were several limitations in our study. i) Sample sizes of *C. propinquum* and *C. simulans* clinical isolates were small. This was because, despite our efforts, we could not find additional isolates. ii) *C. pseudodiphtheriticum* and *C. amycolatum*, which other reports have shown to be common non-diphtheriae corynebacteria [35], were not available for this study; this might have been due to differences in the geographical distribution of non-diphtheriae corynebacteria.

Despite these limitations, our study has some strengths. First, the HRM method can identify and differentiate between *C. striatum*, *C. propinquum*, and *C. simulans* directly from clinical sputum samples without isolating and culturing the pathogens. This meets the need for rapid diagnosis and, more importantly, yields high diagnostic accuracy in culture-negative samples. Second, this assay could potentially be applied to recognize novel or unusual *Corynebacterium* spp. in clinical specimens by targeting single loci, with no need to design new assays; this is supported by previous research [45]. Third, this technique produced results in 2 h with no need for gel electrophoresis of the PCR products, avoiding sample cross-contamination. Finally, HRM analysis is a cost-effective method using common and widely available reagents and equipment, making it especially suitable for resource-limited settings.

## Conclusions

In conclusion, the HRM assay described in this study is the first developed for the detection and identification of *C. striatum*, *C. propinquum,* and *C. simulans* from pure cultures and clinical specimens. It outperformed the culture method, proving to be capable of detecting many samples the culture method missed. The present study demonstrated this HRM assay to be a simple, rapid, accurate, sensitive, specific, and cost-effective diagnostic approach. The HRM assay is a promising alternative for identifying *Corynebacterium* spp., effectively complementing current methods used in clinical microbiology laboratories, and has the potential to contribute to early diagnosis and epidemiological surveillance.

## Supplementary Information

**Additional file 1.** Bacterial strains used in this study.

**Additional file 2.** Representative results of clinical isolates tested by high-resolution melting graphs

## Data Availability

All data generated or analysed during this study are included in this published article [and its supplementary information files].

## Abbreviations

HRM: high-resolution melting
MCDA: multiple cross-displacement amplification
TP: true positive
TN: true negative
FP: false positive
FN: false negative
