# Supplementary material for: Direct detection of Corynebacterium striatum, Corynebacterium propinquum, and Corynebacterium simulans in sputum samples by high-resolution melt curve analysis
Source: BMC Infect Dis. 2021 Jan 7;21:21. doi: 10.1186/s12879-020-05633-z (PMC7788810; doi:10.1186/s12879-020-05633-z)
Supplement: Supplementary file 2 — Additional file 2. Representative results of clinical isolates tested by high-resolution melting graphs [file 12879_2020_5633_MOESM2_ESM.pdf]

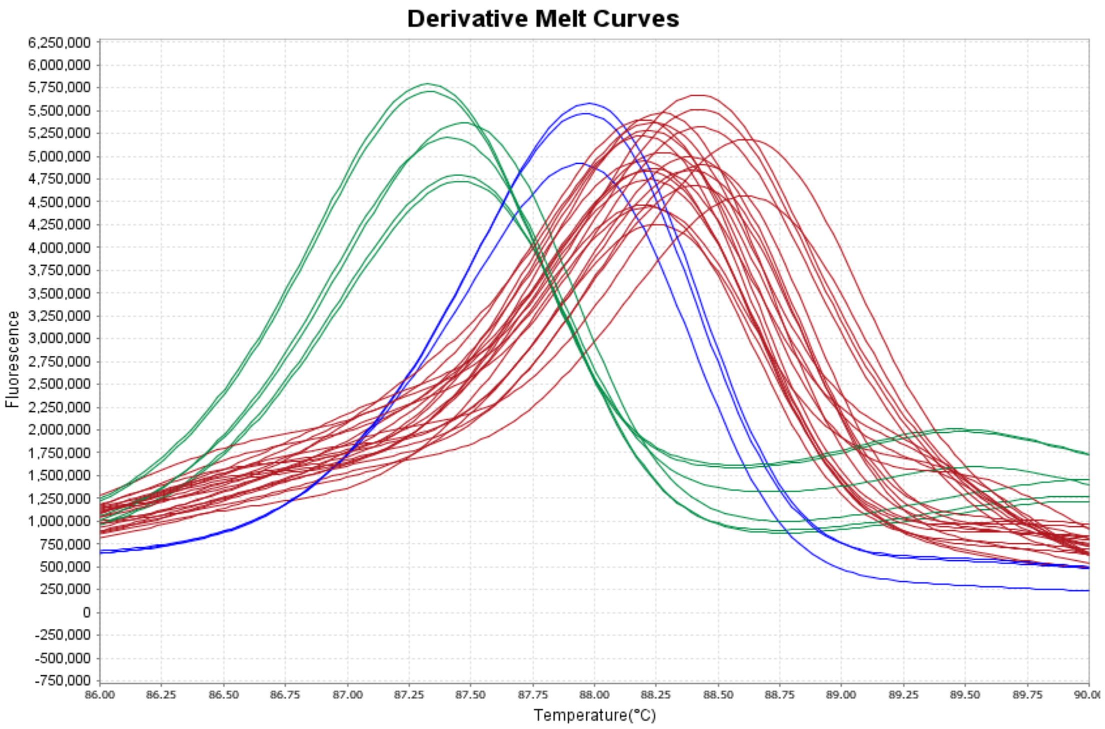

Additional file 2. Representative results of clinical isolates tested by High resolution melting graphs.
